# Clinical Value of Neutrophil Gelatinase-Associated Lipocalin as a Non-Invasive Biomarker for Intestinal Ulcer in Behçet’s Syndrome: A Pilot Study

**DOI:** 10.3390/ijms27073152

**Published:** 2026-03-31

**Authors:** Jing-Fen Ye, Yi-Xuan Zhang, Dan Hu, Jian-Fei Cai, Yu-Xin Liu, Li-Yang Zhang, Jun Zou, Jian-Long Guan

**Affiliations:** Department of Rheumatology and Immunology, Huadong Hospital Affiliated to Fudan University, Shanghai 200040, China; afenningxing@126.com (J.-F.Y.);

**Keywords:** intestinal Behçet’s syndrome, neutrophil gelatinase-associated lipocalin2, biomarker

## Abstract

Intestinal Behçet’s syndrome (BS) is a severe phenotype associated with high morbidity and mortality. Early identification of intestinal involvement in BS remains clinically challenging due to the lack of reliable biomarkers. Neutrophil gelatinase-associated lipocalin (NGAL) is abundantly secreted during intestinal inflammation and has been recognized as a promising inflammatory biomarker. This study was designed to investigate the clinical value of NGAL for predicting intestinal involvement in BS patients. BS patients who underwent colonoscopy for suspected intestinal lesions were enrolled and classified into intestinal BS and non-intestinal BS groups. Immunohistochemistry was performed to compare colonic mucosal NGAL expression among intestinal BS, non-intestinal BS, inflammatory bowel disease (IBD) patients, and healthy controls. Serum and fecal NGAL levels were also measured in intestinal BS, non-intestinal BS, and healthy controls. Intestinal mucosal NGAL expression was significantly elevated in both intestinal BS and IBD patients, with no significant difference between the two groups. Serum and fecal NGAL levels were both higher in intestinal BS patients than in healthy controls. Notably, only fecal NGAL was significantly increased in intestinal BS compared to non-intestinal BS (*p* < 0.001). Multivariate logistic regression analysis identified fecal NGAL as an independent predictive factor for intestinal involvement in BS (OR = 1.093, 95% CI: 1.017–1.174, *p* = 0.015), with superior predictive performance over conventional inflammatory markers. In conclusion, fecal NGAL serves as a non-invasive and promising biomarker for risk stratification of intestinal involvement in BS patients. Further large-scale prospective studies are required to verify these preliminary results.

## 1. Introduction

Behçet’s syndrome (BS) is a variable vessel vasculitis (VVV) of unknown etiology that may affect both arterial and venous vessels, leading to heterogeneous clinical manifestations. Previous cluster analysis by our group identified eight distinct clinical phenotypes within VVV, including mucocutaneous, gastrointestinal, articular, ocular, cardiovascular, neurological, vascular, and hematological involvement [1]. Each phenotype is characterized by distinct clinical features, therapeutic strategies, and prognoses. Mucocutaneous lesions, regarded as the fundamental phenotype of BS, often precede subsequent major organ involvement. Intestinal BS represents a severe subtype of BS associated with high mortality and morbidity. Approximately 22.16% of patients with systemic BS develop intestinal involvement within 4–6 years after disease onset, and about 61.54% of affected patients lack obvious gastrointestinal symptoms [2]. In addition, the gastrointestinal and extra-intestinal manifestations of intestinal BS overlap considerably with those of inflammatory bowel disease (IBD), including Crohn’s disease (CD) and ulcerative colitis (UC) [3]. Owing to the insidious onset of gastrointestinal symptoms, the absence of well-accepted specific laboratory biomarkers, and nonspecific pathological features, the diagnosis of intestinal BS is often delayed, resulting in late initiation of targeted therapy. Therefore, it remains clinically challenging to identify and manage gastrointestinal lesions in BS patients before irreversible organ damage occurs. Currently, colonoscopy is indispensable for evaluating gastrointestinal involvement in BS [4]. Nevertheless, colonoscopy is invasive, poorly tolerated, time-consuming, and expensive, and carries inherent risks such as intestinal bleeding or perforation, particularly in patients with active intestinal BS, limiting its utility as a routine screening tool. Although conventional inflammatory markers, including erythrocyte sedimentation rate (ESR) and C-reactive protein (CRP), have been suggested as potential predictors of intestinal ulcers in BS patients [5], these markers lack sufficient sensitivity and specificity for diagnosis and monitoring treatment response. Accordingly, there is an urgent need for simple, noninvasive, and easily repeatable biomarkers suitable for screening and real-time assessment of intestinal involvement in BS patients.

Recent studies have linked neutrophil aggregation and inflammatory activation to BS-mediated inflammatory organ damage. Infliximab-induced disease remission has been reported to rely on the rapid inhibition of neutrophil recruitment [6]. Accordingly, several neutrophil-related markers have been identified as promising biomarkers for vascular involvement in BS [7,8]. Neutrophil gelatinase-associated lipocalin (NGAL; lipocalin-2) is a bacteriostatic protein originally isolated from and named after neutrophils. It is stored in the granules of mature neutrophils and released upon cellular activation. In metabolic inflammation, elevated NGAL expression promotes inflammatory responses by driving the differentiation and recruitment of Th17 and Th2 lymphocytes via the release of proinflammatory cytokines, establishing NGAL as a pleiotropic mediator in diverse inflammatory processes [9]. NGAL is a secreted glycoprotein that exhibits characteristics of an acute-phase protein. Beyond neutrophils, NGAL is also expressed by respiratory and intestinal epithelial cells, endothelial cells, and renal tubular cells during inflammation and tissue injury, representing an attractive biomarker for inflammation, ischemia, infection, and renal damage [10]. In response to renal tubular injury, NGAL expression is markedly upregulated. Increased NGAL levels can be detected within hours of tubular insult and demonstrate superior performance over serum creatinine for the early prediction of renal injury [11]. NGAL is strongly induced by a broad spectrum of proinflammatory stimuli, including IL-1β, IL-22, and Toll-like receptor (TLR) activation, and is secreted at high concentrations into the intestinal lumen [12]. Following gastrointestinal injury, bacterial infection, or intestinal inflammation, NGAL expression is significantly enhanced, leading to high mucosal and fecal levels of this protein. Given its easy detectability in multiple body fluids, NGAL has been explored in recent years as a key indicator for evaluating the severity of intestinal inflammatory conditions [13]. Despite these findings, the clinical utility of NGAL as a non-invasive biomarker for intestinal involvement in BS remains largely unclear. Therefore, this study aimed to determine whether serum or fecal NGAL levels are associated with intestinal involvement in BS, and to explore its potential as a noninvasive indicator for early identification and monitoring of gastrointestinal lesions in this population.

## 2. Results

### 2.1. Participant Characteristics

A total of 66 patients with Behçet’s syndrome (46 intestinal BS and 20 non-intestinal BS), 5 patients with IBD, and 20 healthy controls were enrolled. Baseline demographic and clinical characteristics are presented in Table 1. Sex, age, and disease duration were comparable among all groups (all *p* > 0.05). Serum and fecal samples were obtained from all BS patients and healthy controls, and 5 subjects from each group were included for immunohistochemical analysis. The frequencies of systemic manifestations including oral ulcers, genital ulcers, skin, ocular, cardiovascular, articular, neurological, and hematological involvement were similar between intestinal and non-intestinal BS groups. However, gastrointestinal manifestations were significantly more common in intestinal BS patients (*p* = 0.036). Furthermore, ESR and CRP, and fecal calprotectin levels were significantly higher in the intestinal BS group than in the non-intestinal BS group (*p* < 0.05).

### 2.2. Neutrophil Gelatinase-Associated Lipocalin Protein Expression in Colonic Biopsies

**Figure 1:**
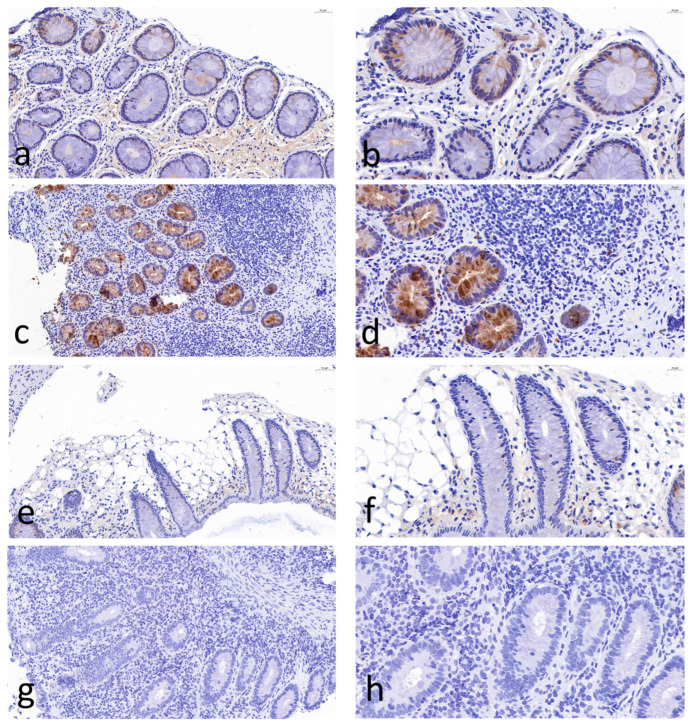
Immunohistochemical staining of neutrophil gelatinase-associated lipocalin (NGAL) in human colon biopsies from patients with inflammatory bowel disease (IBD) (**a**,**b**), intestinal Behcet’s syndrome (BS) (**c**,**d**), non-intestinal BS (**e**,**f**), and healthy controls (**g**,**h**). Images are representative of independent experiments. immunohistochemical result showed intense staining of NGAL in infiltrating inflammatory cell and enterocytes of the ileocolonic mucosal tissue in IBD and intestinal BS, whereas weak staining was observed in non-intestinal BS and healthy controls. Original magnification: ×20 (**left** panels) and ×40 (**right** panels).

**Figure 2:**
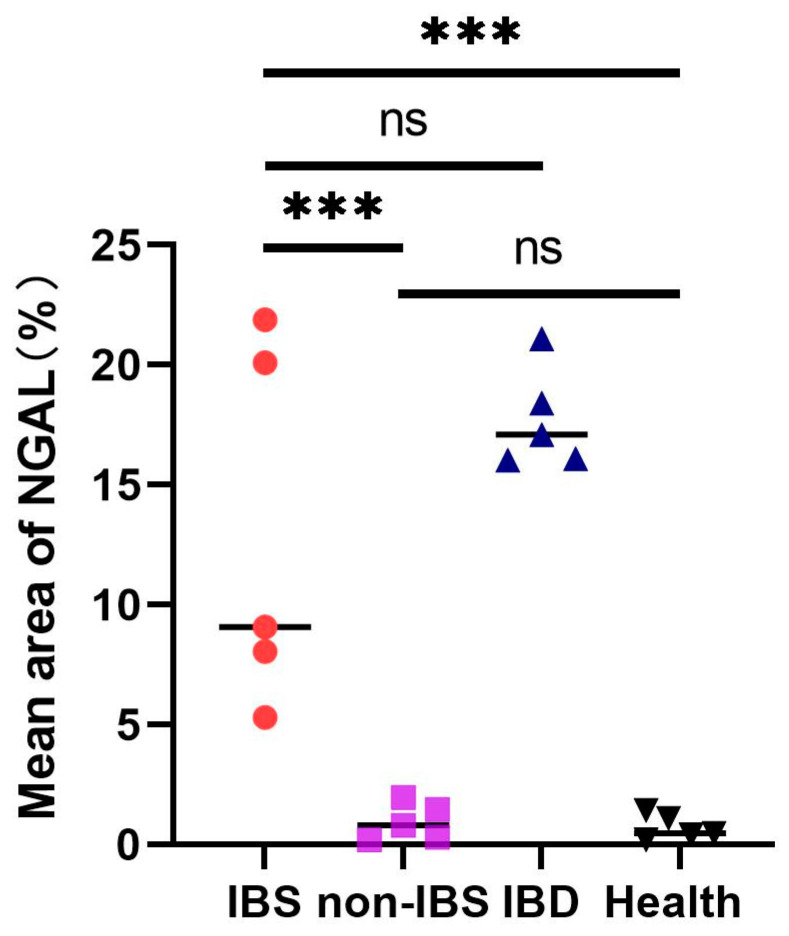
The mean area of neutrophil gelatinase-associated lipocalin (NGAL)-positive staining was evaluated using Fiji (Image J 1.54r, National Institutes of Health, Bethesda, MD, USA) software in ileocolonic biopsies from intestinal Behcet’s syndrome (IBS, red circles), non-IBS (purple squares), inflammatory bowel disease (IBD, blue triangles), and healthy control (Health, black inverted triangles) groups. IBS groups exhibited significantly higher NGAL mean area (*** *p* < 0.001) compared with non-IBS and healthy controls, while no significant differences were found between IBS and IBD, or between non-IBS and healthy groups (ns). Statistical analysis was performed by *t*-test.

Figure 1 shows immunohistochemical (IHC) staining of neutrophil gelatinase-associated lipocalin (NGAL) in the colon tissues from patients with inflammtory bowel disease (IBD) (Figure 1a,b), intestinal Behcet’s syndrome (BS) (Figure 1c,d), non-intestinal BS (Figure 1e,f), and healthy controls (Figure 1g,h). IHC analysis revealed significantly increased mucosal NGAL protein in intestinal BS and IBD patients. for protein. In contrast, NGAL staining in endoscopic pinch biopsies from non-intestinal BS patients was faint and comparable to that in healthy controls. In intestinal BS and IBD patients, NGAL protein was localized to both infiltrating inflammatory cells and mucosal epithelial cells. A semi-quantitative scoring method was applied to objectively assess NGAL staining intensity using Fiji (ImageJ 1.54r, National Institutes of Health, Bethesda, MD, USA) (Figure 2). Intestinal BS patients exhibited a significantly higher mean area of NGAL protein expression compared with non-intestinal BS patients (12.8% ± 7.66% vs. 0.4% ± 0.55%, *p* < 0.001) and healthy controls (12.8% ± 7.66% vs. 1.2% ± 1.30%, *p* < 0.001). Notably, no significant difference was detected between intestinal BS and IBD groups (12.8% ± 7.66% vs. 17.60% ± 2.07%, *p* = 0.213), suggesting a shared inflammatory signature in these conditions. Similarly, non-intestinal BS patients showed comparable NGAL expression levels to healthy controls (0.4% ± 0.55% vs. 1.2% ± 1.30%, *p* = 0.242).

### 2.3. Serum Levels of Neutrophil Gelatinase-Associated Lipocalin 

**Figure 3:**
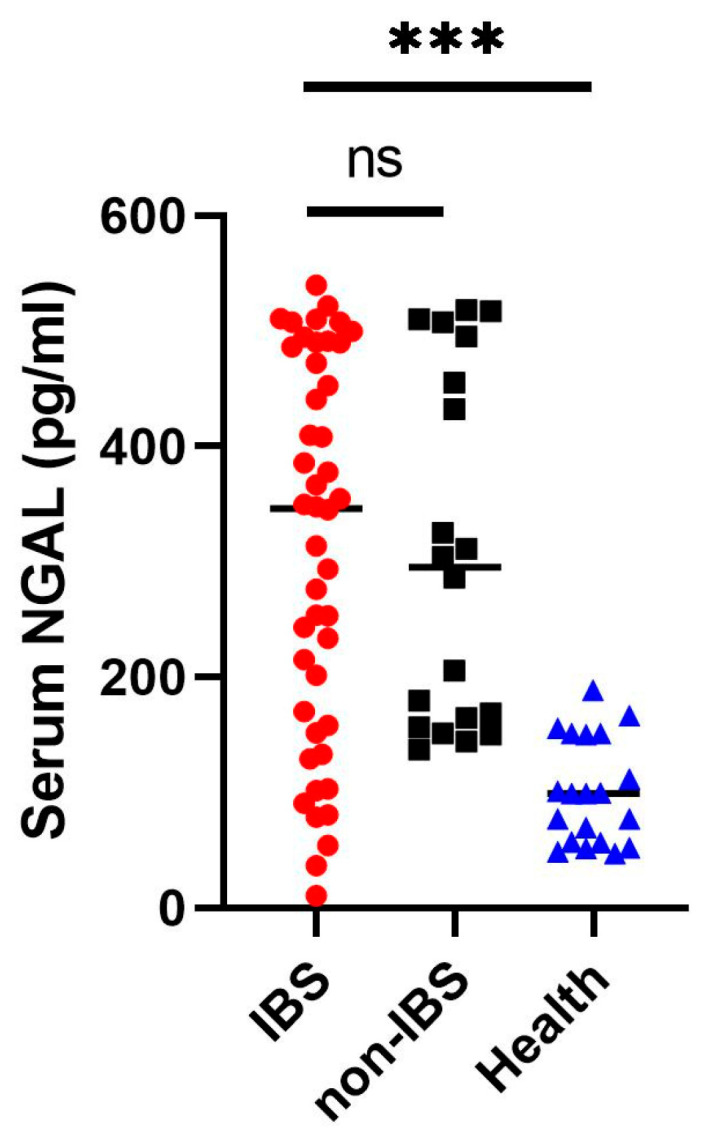
Comparison of serum neutrophil gelatinase-associated lipocalin (NGAL) concentrations between intestinal Behcet’s syndrome (IBS, red circles), non-IBS (black squares), and healthy control (Health, blue triangles). Serum NGAL levels were significantly higher in IBS patients than in healthy controls (*** *p* < 0.001). No statistically significant difference was found in serum NGAL levels between IBS and non-IBS patients (ns). Medians are indicated by horizontal lines.

Given that the increased mucosal neutrophil gelatinase-associated lipocalin (NGAL) expression in intestinal Behcet’s syndrome (BS) patients was statistically significant only when compared with non-intestinal BS patients and healthy individuals, but not with inflammatory bowel disease (IBD) patients, NGAL may represent a marker of intrinsic intestinal damage in the BS population. We therefore measured serum NGAL levels in patients with intestinal BS, non-intestinal BS, and healthy controls. Serum NGAL levels were significantly elevated in intestinal BS patients [n = 46, median 346.5 (IQR 156.75–487.00)] compared with healthy controls [n = 20, median 99 (IQR 56.75–151.00), *p* < 0.001], whereas no significant difference was observed between intestinal BS and non-intestinal BS patients [n = 20, median 295 (IQR 158.75–485.25), *p* = 0.929] (Figure 3).

### 2.4. Fecal Neutrophil Gelatinase-Associated Lipocalin Levels Are Increased in Behcet’s Syndrome 

**Figure 4:**
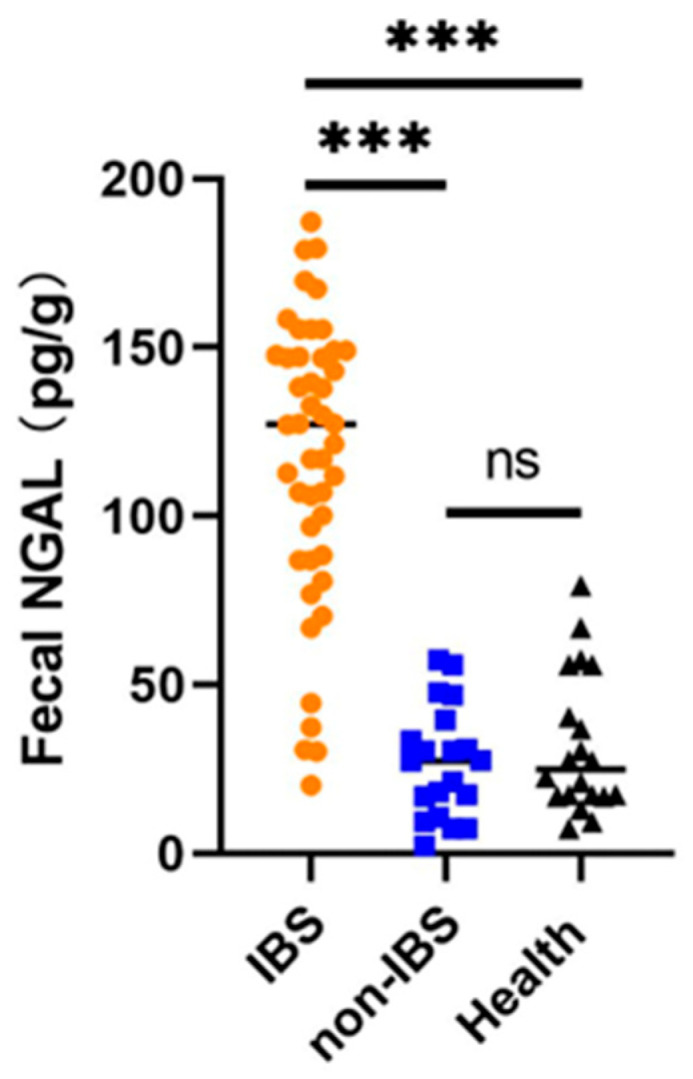
Fecal neutrophil gelatinase-associated lipocalin (NGAL) levels in patients with intestinal Behcet’s syndrome (IBS, orange circles), non-IBS (blue squares), and healthy control (Health, black triangles). Fecal NGAL levels were significantly higher in IBS patients (orange) than in non-IBS patients (blue) and healthy controls (black) (*** *p* < 0.001). No significant difference was observed between non-IBS and healthy groups (ns). Medians are indicated by horizontal lines.

To further validate neutrophil gelatinase-associated lipocalin (NGAL) as a non-invasive biomarker for intestinal Behcet’s syndrome (BS) in clinical practice. Fecal NGAL levels were compared among intestinal BS patients, non-intestinal BS patients, and healthy controls. As shown in Figure 4, fecal NGAL concentrations in the intestinal BS group were significantly higher than those in both the non-intestinal BS group (127.00 [87.75–148.25] pg/g vs. 27.50 [12.50–48.25] pg/g, *p* < 0.001) and the healthy control (127.00 [87.75–148.25] pg/g vs. 25.00 [17.00–52.00] pg/g, *p* < 0.001). In contrast, no significant difference in fecal NGAL levels was observed between the non-intestinal BS group and healthy controls (27.50 [12.50–48.25] pg/g vs. 25.00 [17.00–52.00] pg/g, *p* = 0.4075). These findings suggest that elevated fecal NGAL may reflect intestinal inflammation or epithelial injury in the pathogenesis of intestinal BS.

### 2.5. Predictors of Intestinal Behcet’s Syndrome 

To identify whether fecal NGAL is a useful surrogate marker for predicting intestinal involvement among BS patients. We next performed multivariate analysis. Gastrointestinal symptom, which was identified as a factor associated with intestinal BS, according to the chi-square test result, was included in the multivariate analysis. Laboratory markers indicating inflammation, such as ESR, CRP, and fecal calprotectin, were also adjusted. In multivariate analysis, the factor predictive for intestinal involvement in patients with BS was fecal NGAL OR = 1.093, 95% CI: 1.017–1.174, *p* = 0.015), indicating that each unit increase in fecal NGAL was associated with a 9.3 increase in the risk of intestinal BS. (Table 2).

## 3. Discussion

Behçet’s syndrome (BS), presenting with gastrointestinal ulcer, may suffer from a range of conditions, including intestinal obstruction, bowel perforation, or severe hematochezia, which necessitates surgical treatment [14]. Therefore, early diagnosis and evaluation of intestinal BS are critically important for guiding treatment and improving prognosis. Neutrophil gelatinase-associated lipocalin (NGAL) increases in inflamed colonic epithelium, has immunoregulatory functions, and reflects neutrophil activation [15]. In this study, we investigated the use of NGAL in the diagnosis of intestinal BS.

As an exploratory study, we investigated NGAL protein expression using immunohistochemistry in 20 colonic mucosal biopsies, aiming to validate the clinical utility of NGAL in intestinal BS. Our results demonstrate elevated mucosal NGAL expression in patients with intestinal BS compared with both healthy controls and patients with non-intestinal BS. Previous studies have reported significantly increased NGAL levels in the intestinal epithelium of patients with active inflammatory bowel disease (IBD) relative to controls [16,17]. NGAL has also been shown to reliably distinguish IBD from functional bowel disorders such as irritable bowel syndrome [18]. To date, however, no study has directly compared mucosal NGAL expression between intestinal BS and IBD, which represents a key novelty of the present work. In clinical practice, IBD is the most important differential diagnosis for intestinal BS. Nevertheless, our semi-quantitative analysis of mucosal NGAL expression revealed no significant difference between intestinal BS and IBD groups. These immunohistochemical findings indicate that mucosal NGAL expression has limited diagnostic value for differentiating intestinal BS from IBD. Therefore, NGAL is not a specific marker for intestinal BS, as fecal NGAL levels were significantly elevated in active Ulcerative Colitis (UC) (6.05 [3.6–15.1] mg/kg), active Crohn’s disease (CD) (4.9 [1.5–7.7] mg/kg), and infectious enterocolitis (2.7 [1.4–5.6] mg/kg) compared with inactive IBD and healthy controls (0.3 [0.1–0.4] mg/kg) [18]. Furthermore, Bakke et al. reported that Lipocalin-2 (LCN2) expression was upregulated in active collagenous colitis (fold change = 6.47, log_2_ FC = 2.69, *p* = 0.005) relative to healthy controls. Collectively, the above findings indicate that increased NGAL expression reflects a general inflammatory response rather than a BS-specific pathological change [13].

To further verify its clinical significance, serum NGAL levels were measured in patients with intestinal BS, patients with systemic BS without endoscopic evidence of intestinal ulcers, and healthy control subjects. We found that serum NGAL levels were elevated in all patients with BS, regardless of intestinal involvement. These results are consistent with previous reports [19,20]. Previous studies have suggested that serum NGAL serves as a valuable clinical marker of intestinal inflammation and has emerged as a promising biomarker with excellent diagnostic performance for identifying IBD [21]. However, our findings indicate that serum NGAL does not specifically reflect intestinal inflammation in patients with BS. Celik F. et al. reported that serum NGAL is a useful clinical marker for the ocular subtype of BS and correlates with disease activity [19,20]. To the best of our knowledge, no prior study has compared serum NGAL levels across different clinical subtypes of BS. The present study is the first to evaluate serum NGAL levels in patients with the intestinal BS subtype. Nevertheless, our results demonstrate that serum NGAL has no predictive value for identifying patients with systemic BS who subsequently develop intestinal involvement.

In intestinal BS biopsies, we show increased NGAL protein expression. On this background, we hypothesized that fecal NGAL may be a predictive biomarker of BS with endoscopic evidence of intestinal ulcers, but this remains to be proven. We further measured NGAL levels in fecal samples. In our study, fecal NGAL measurements showed several promising results for the diagnosis of patients with intestinal BS. The statistical results show that the median fecal NGAL level in patients with intestinal BS was significantly higher than in BS patients without intestinal involvement and in healthy controls. It has been previously reported that fecal NGAL amounts are increased in other inflammatory diseases such as IBD [12] and multiple sclerosis [22]. In addition, we performed multivariate logistic regression analysis to identify independent predictors of intestinal involvement in BS, adjusting for potential confounders including age, sex, systemic inflammation [erythrocyte sedimentation rate (ESR) and C-reactive protein (CRP)], as well as fecal calprotectin for intestinal mucosal inflammation. The results revealed that fecal NGAL was an independent risk factor for intestinal BS (OR = 1.093, 95% CI: 1.017–1.174, *p* = 0.015), with each unit increase in fecal NGAL associated with a 9.3 elevated risk of intestinal involvement. Previous studies have suggested fecal calprotectin, ESR, and CRP as conventional markers for discriminating intestinal involvement in BS [5]. Our comparative analysis further demonstrated that fecal NGAL outperformed these conventional markers in the multivariate model. Altogether, our findings suggest that fecal NGAL may serve as a complementary biomarker to conventional monitoring tools for identifying BS patients at high risk of intestinal involvement to guide selective colonoscopy screening, thereby ensuring accurate and timely diagnosis of intestinal BS. Fecal NGAL levels represent a low-cost, easily measurable biomarker for stratifying the risk of prevalent intestinal involvement in patients with BS, with potential to reduce unnecessary invasive procedures and optimize clinical resource allocation.

There are several limitations in our study. First, it was a single-center pilot project. A small sample size may not fully capture the comprehensive characteristics of intestinal BS, which limits the generalizability and statistical power of the results when analyzing the predictive value of NGAL. Second, we could not assess NGAL gene expression due to the unavailability of examinations at our hospital and financial constraints. Thirdly, only baseline serum and feces NGAL levels were assessed, without evaluation of dynamic changes or parallel feces measurements, which might have reduced the biomarker’s sensitivity and discriminative capacity. Additionally, whether the observed increase in NGAL levels is a cause or a consequence of the disorder remains to be determined and warrants further investigation. Future studies are needed to clarify the role of NGAL in this disorder.

## 4. Materials and Methods

### 4.1. Patients and Clinical Samples

Study subjects were retrospectively enrolled from patients with systemic Behçet’s syndrome (BS) who underwent colonoscopy for the evaluation of intestinal involvement. All BS patients were subsequently divided into two subgroups based on colonoscopic findings: intestinal BS and non-intestinal BS. Control groups included age- and gender-matched inpatients diagnosed with inflammatory bowel disease (IBD) as well as healthy volunteers who underwent routine colonoscopy with normal findings. Endoscopic pinch biopsies were obtained from the maximally inflamed mucosal lesions of BS patients and IBD patients. For healthy controls, biopsies were collected from non-inflamed mucosal sites. All tissue specimens were formalin-fixed, paraffin-embedded (FFPE), and sectioned for routine histological and immunohistochemical (IHC) analyses. Peripheral blood was collected from all untreated BS patients, IBD patients, and healthy controls prior to any medical intervention. Serum was separated by centrifugation and stored at −80 °C until analysis. Fecal samples were collected from all subjects before endoscopy. Specifically, fecal specimens from BS patients, IBD patients, and healthy controls were collected and stored at −80 °C without additional preservatives until measurement of NGAL levels.

### 4.2. Histology and Immunohistochemistry

Histology and immunohistochemistry (IHC) examinations were performed on 20 randomly selected biopsies, including healthy controls, non-intestinal BS, intestinal BS, and IBD (5 from each group). Normal or inflammatory findings were confirmed on haematoxylin and eosin-stained slides by two experienced pathologists before the sample was included in the analysis. Primary antibody, rabbit polyclonal anti-human NGAL (antibody 44085S; Cell Signaling Technology (CST), Danvers, MA, USA), was diluted 1:3000. Secondary antibody was obtained from Abcam (Cat# AB205718, rabbit/goat; Abcam, Cambridge, UK), and detection was performed using diaminobenzidine (3–5 min) + chromogen (Dako, Glostrup, Denmark). For quantitative analysis of IHC staining, three non-overlapping fields were randomly selected from each section under high-power magnification (×400). The mean NGAL positive staining area was measured using Fiji (ImageJ 1.54r, National Institutes of Health, Bethesda, MD, USA) software, and the average value was used for statistical analysis.

### 4.3. Blood Sampling and Analysis

Serum samples were obtained according to standardized protocols in the Hospital at the time of recruitment. In total, NGAL was measured in 86 samples from 66 BS (46 with intestinal involvement and 20 without) and 20 healthy controls. NGAL in serum was quantified with a sandwich ELISA Kit (Cat# DY1757; R&D Systems, Minneapolis, MN, USA) according to the manufacturer’s instructions. Briefly, we first prepared the plates and reagents. Next, add 100 μL of the sample or standard to the Reagent Diluent. The plates were covered with an adhesive strip and incubated for 2 h at room temperature. Then, it was washed three times with Wash Buffer (300 μL), and 100 μL of Detection Antibody was added to each well. After an incubation step of 2 h (room temperature), the plate was repeatedly washed three times. At the end, 50 μL of the working dilution of Streptavidin-HRP B was added, and the plate was incubated for 20 min at room temperature, with the plate covered to avoid direct light. Finally, after adding 50 μL of Sulfuric Acid Stop Solution, all samples were quantified by measuring optical density at 450 nm.

### 4.4. Processing of Fecal Samples and Fecal NGAL Measurement

Stool samples were obtained 3 days before bowel preparation. The fecal sample was obtained from the research subject who provided the serum sample. Using a disposable stool sampler (For Routine Fecal Examination), 5 g of fecal specimen was collected and stored at −80 °C before extraction. The thawed stool sample (1 g) was mixed with 9 mL of phosphate-buffered saline (PBS) and pipetted for 1 min. Then, it was centrifuged at 3000 rpm for 20 min, allowed to stand for 10 min, and the supernatant was transferred to a clean tube for detection. NGAL in fecal samples were quantified using a sandwich ELISA Kit (Cat# DY1757; R&D Systems, Minneapolis, MN, USA). The detection steps are the same as for serum.

### 4.5. Statistical Analysis

Continuous variables were compared using the Mann–Whitney U-test or Student’s *t*-test as appropriate and were presented as the median (range) or the mean ± standard deviation in this paper. Categorical variables were compared using the chi-square test and presented as proportions. Owing to the relatively small sample size, a reliable optimal cutoff value could not be determined by receiver operating characteristic (ROC) curve analysis. Therefore, the biomarker was included as a continuous variable in binary logistic regression analysis to evaluate its independent association with intestinal involvement in BS patients. Age, gender, and inflammatory markers were adjusted by “enter” method of statistical program as covariates in regression model. Calculations were performed using SPSS version 19.0 and GraphPad Prism version 9.0. A *p* value < 0.05 was considered significant.

### 4.6. Ethical Considerations

All patients gave informed written informed consent to participate in this study. This study involving humans were approved by the Medical Ethics Committee of Huadong Hospital affiliated to Fudan University (identifier 2023K098).

## 5. Conclusions

Collectively, the current evidence from both the literature and our study demonstrates that NGAL is significantly upregulated in the inflamed intestinal mucosa of BS patients with intestinal involvement, and this elevation is readily reflected in fecal samples. Notably, fecal NGAL levels hold promise as a cost-effective, non-invasive biomarker for identifying BS patients at high risk of prevalent intestinal involvement. Nevertheless, further validation through large-scale prospective studies is warranted to confirm these findings and establish more definitive clinical conclusions.

## Figures and Tables

**Table 1:** Characteristics of subjects included in immunohistochemical, serum, and fecal neutrophil gelatinase-associated lipocalin analyses.

	Health Controls	Intestinal BS	Non-Intestinal BS	IBD	*p*
Number of samples					
Total, n	20	46	20	5	
IHC samples, n	5	5	5	5	-
Serum samples, n	20	46	20	-	-
Fecal samples, n	20	46	20	-	-
Demographic characteristic					
Gender, Female (Male)	10 (10)	25 (21)	9 (11)	2 (3)	N.S.
Age, years	37.30 ± 12.07	38.89 ± 13.55	34.05 ± 11.73	33.40 ± 6.66	N.S.
Disease duration (months)	-	44.00 ± 35.67	43.13 ± 31.18	44 (24–63)	N.S.
Treatment	-	None	None	None	-
systemic symptom	-			-	
Oral ulcer n (%)	-	45 (97.83)	18 (90.00)	-	0.216
Genital ulcer n (%)	-	32 (69.57)	11 (55.00)	-	0.194
Skin lesion n (%)	-	20 (43.48)	10 (50.00)	-	0.542
Ocular n (%)	-	1 (2.17)	2 (2.00)	-	0.216
Cardiovascular n (%)	-	2 (4.35)	0 (0)	-	0.483
Arthritis n (%)	-	8 (17.39)	1 (5.00)	-	0.171
Nervous n (%)	-	1 (2.17)	0 (0)	-	0.697
Hematological n (%)	-	3 (6.52)	1 (5.00)	-	0.667
Gastrointestinal manifestations n (%)	-	24 (52.17)	5 (25.00)	5 (100)	* 0.036
laboratory index					
ESR (mm/h)	7 (3–11)	20 (12–34.00)	10 (4–19)	45 (33–78)	* 0.010
CRP (mg/L)	6 (3–9)	15 (8.00–31)	8 (4–12)	44 (18–67)	* 0.005
Fecal calprotectin (ug/g)	11 (5–44)	84 (43–110)	18 (8–48)	98 (51–316)	* 0.005

BS, Behçet’s syndrome; IBD, Inflammatory bowel disease; IHC, immunohistochemistry; N.S., not significant; ESR, erythrocyte sedimentation rate; CRP, C-reactive protein. * *p* < 0.05 Intestinal BS vs. Non-intestinal BS. n, representing number of items.

**Table 2:** Multivariate logistic regression analysis for predictors of intestinal involvement in Behcet’s syndrome.

	B	SE	*p*	OR (95% CI)
CRP	−0.26	0.056	0.639	0.974 (0.873–1.087)
ESR	0.32	0.066	0.629	1.033 (0.906–1.176)
Fecal calprotectin	0.28	0.019	0.145	1.028 (0.991–1.067)
Fecal NGAL	0.89	0.037	* 0.015	1.093 (1.017–1.174)
Gastrointestinal manifestations	−3.162	1.669	0.058	0.042 (0.002–1.116)

CRP, C-reactive protein; ESR, erythrocyte sedimentation rate; NGAL, neutrophil gelati nase associated lipocalin; OR, odds ratio; CI, confidence interval. Continuous variables were entered into the model without dichotomization. * *p* < 0.05. B: Beta coefficient; SE: Standard Error.

## Data Availability

The original contributions presented in this study are included in the article. Further inquiries can be directed to the corresponding author.
